# Metallurgical and Mechanical Properties of Stellite 6 Deposition Developed Through Friction Surfacing Technique

**DOI:** 10.3390/ma18051003

**Published:** 2025-02-25

**Authors:** Mohammad Faseeulla Khan, Ramachandran Damodaram, Hussain Altammar, Gangaraju Manogna Karthik

**Affiliations:** 1 Department of Mechanical Engineering, College of Engineering, King Faisal University, Al-Ahsa 31982, Saudi Arabia; fmohammad@kfu.edu.sa (M.F.K.); haltammar@kfu.edu.sa (H.A.); 2 Department of Mechanical Engineering, Sri Sivasubramaniya Nadar College of Engineering, Chennai 603110, India; 3 Department of Mechanical Engineering, Indian Institute of Technology (BHU), Varanasi 221005, India

**Keywords:** Stellite 6, friction surfacing, plasma transferred arc, coatings, dilution, wear

## Abstract

Friction surfacing (FS) is a solid-state process for depositing metallurgically bonded coatings for corrosion and wear protection. It is particularly attractive for depositing coatings in materials that are difficult to fusion deposit. Stellite 6 is one such material, which is widely used as a protective coating on steel structures to combat wear and corrosion. In the current study, Stellite 6 was successfully friction-surfaced on low carbon steel plates without using any preheating. The microstructures and wear behavior of Stellite 6 coatings produced using FS were comparatively investigated with those produced using the plasma transferred arc (PTA) process. The PTA coatings showed a cast microstructure consisting of γ-dendrites and an inter-dendritic carbide network. On the other hand, the FS coatings showed a wrought microstructure with dynamically recrystallized grains and fine, uniformly distributed carbide particles. The FS coatings also showed uniform composition across the coating thickness and were undiluted, while the PTA coatings showed significant dilution as well as strong local variations in chemistry. The FS coatings exhibited a 22% increase in hardness (550 HV) compared to the PTA coatings (450 HV). Pin-on-disc dry sliding wear tests showed that the FS coatings (1.205 mm^3^) were more wear resistant compared to the PTA coatings (6.005 mm^3^), highlighting their superior mechanical performance. This study uniquely demonstrates the feasibility of depositing Stellite 6 coatings using FS without the need for preheating or post-deposition heat treatments, while achieving superior microstructural refinement, hardness, and wear resistance compared to conventional PTA coatings.

## 1. Introduction

Low carbon steel (LCS) is a mixture of a low ratio of carbon to iron; it contains less than 0.3% of carbon, which results in no improvement in strength, hardness, and wear properties with heat treatment. These steels are relatively weak and soft but are ductile [1]. Due to its softness and ductility, LCS is mainly used in machinery and welding parts at a low cost; for example, it is used in automobile body parts, food cans, bridge components, and structural components like I-beams [1]. However, due to their low hardness and poor wear resistance, LCS requires efficient surface engineering procedures to increase its wear resistance [2].

Stellite 6 is a cobalt-based alloy that consists of complex carbides. It performs exceptionally well in wear, erosion, and corrosion resistance [3,4,5,6,7]. Stellite 6 is used in high-temperature and high-pressure scenarios. Due to its good corrosion resistance, it is primarily used in light water nuclear reactors and valve applications [5,8,9,10]. Along with preventing erosion and corrosion, it also exhibits excellent wear resistance due to its CoCr alloy matrix. The dendritic carbides present in the CoCr alloy matrix improve the toughness and wear resistance of the Stellite 6 coating [3,4,5,6,7,11,12,13].

Recent studies have investigated various methods to enhance the wear resistance of Stellite 6 coatings. Cinca and Guilemany (2013) demonstrated that cold gas spraying, a solid-state process, effectively deposits Stellite 6 coatings with notable wear resistance under abrasive and sliding conditions [14]. Wei et al. (2024) found that adding titanium and nickel to laser-cladded Stellite 6 coatings led to the formation of TiC particles, improving wear resistance by approximately 13% and significantly enhancing thermal fatigue performance [15]. Felix-Martinez et al. (2024) investigated Stellite 6/WC-6Co composite coatings produced via laser cladding and observed that continuous wave deposition resulted in higher hardness and superior wear resistance at both room and elevated temperatures, attributed to increased diffusion of tungsten and carbon into the matrix [16]. Additionally, research by Manukonda et al. (2024) indicated that incorporating TiC into Stellite 6 coatings on stainless steel substrates via laser cladding significantly improved wear resistance due to the hardening effect of TiC particles [17]. Collectively, these studies highlight the effectiveness of both fusion and solid-state processes, as well as compositional modifications, in enhancing the wear resistance of Stellite 6 coatings.

In short, Stellite 6 coatings are widely used to improve mechanical properties and wear resistance when deposited on various alloys. These coatings can be applied using different techniques, such as gas tungsten arc coating (GTAC), plasma transferred arc (PTA) coating, laser melting, and cladding, etc. [2,9,11,12,18,19,20,21,22,23,24,25]. A comparative study in [9] analyzed various techniques for applying Stellite 6 coatings, including high velocity oxygen fuel (HVOF) spraying (a thermal spraying process) and GTAW. HVOF spraying is commonly used in hydraulic machinery due to its superior performance in water-based corrosion resistance [5,12,22,26,27,28,29,30]. Studies have shown that depositing Stellite 6 coatings using these techniques significantly enhances the wear properties of the base metal while reducing the coefficient of friction. However, these coating techniques involve high temperatures and pressures, making them more suitable for hard and tough alloys.

Friction surfacing (FS) is a solid-state coating technique that applies thick coatings for wear prevention. Unlike fusion-based and thermal spraying processes, FS does not involve melting, thereby eliminating issues related to dilution between the base and weld metal. Fusion-based welding techniques suffer from hot cracking over prolonged periods, leading to material degradation [2]. Therefore, FS provides a more reliable alternative by effectively mitigating these issues, offering significant improvements in wear resistance and durability. This makes FS an ideal method for depositing Stellite 6 coatings onto LCS to enhance wear resistance. Furthermore, FS has gained global acceptance due to its effectiveness [2,24,25,26]. However, limited research exists on FS coatings of Stellite 6 and their wear performance [2].

In the present study, the authors have attempted, for the first time, to understand the significance of FS coatings of Stellite 6 in wear characteristics. Stellite 6 coatings were deposited on LCS using the FS technique, a solid-state coating method, and compared with those produced using the PTA process. To evaluate wear resistance, pin-on-disc dry wear tests were conducted on both FS and PTA coatings, analyzing the coefficient of friction (µ) and wear surface morphology. This study provides new insights into the wear behavior of FS coatings and their microstructural characteristics, which have not been extensively explored in the existing literature. Unlike conventional fusion-based methods, FS offers superior coating integrity with minimal dilution, improving long-term durability. This research advances surface engineering strategies for enhancing the wear resistance of LCS, making FS a viable alternative to traditional techniques.

## 2. Materials and Methods

### 2.1. Coating Process

**Figure 1:**
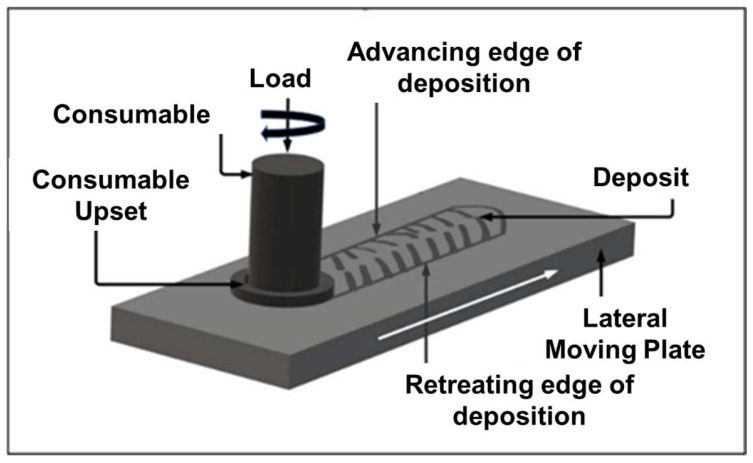
Schematic of the friction surfacing.

Friction surfacing (FS) is a solid-state surface technique where a consumable rod to be deposited as the coating material will be fixed into the rotating chuck/vertical head. The rotating consumable rod will be brought in contact with a stationary substrate under a constant predefined axial load. The consumable rod material is softened because of frictional heating at the rubbing interface between the rod and the substrate. After this, the substrate will be traversed and a constant feed and the softened consumable rod at one end will deposit over the substrate as surface coating. The schematic of the FS is given in Figure 1. In the present work, Stellite 6 was used as the consumable rod material. The consumable rod is 10 mm in diameter and 150 mm in height, obtained through casting. Hot rolled LCS plate of 12 mm thickness is used as substrate material. Before the FS process, the substrate surface was mechanically cleaned to remove oxides, contaminants, and surface irregularities. Then, the substrate was firmly fixed to avoid any movement during the coating process, ensuring uniform deposition. The chemical composition of Stellite 6 (consumable rod) and LCS (substrate) is given in Table 1.

To study and compare the effect of solid-state FS Stellite 6 coating on wear characteristics, a fusion-based surface coating technique, PTA, was used to deposit Stellite 6 coatings. PTA is very similar to the plasma transfer arc welding principle. However, here the objective of the process is to apply coatings rather than joining. In the case of PTA process, Stellite 6 powder was fed into the arc column to obtain the coating. The average grain size of the Stellite 6 powder is 120 ± 10 µm. As there will be significant dilution during PTA process, two layers of coatings, one over the other, were deposited by PTA process. The primary process parameters involved and used for PTA and FS processes are listed in Table 2 and Table 3, respectively.

### 2.2. Microstructural Characterization

For microstructural characterization, the samples were sectioned in the transverse direction from the FS, PTA, and consumable rod. Subsequently, the samples were polished by following standard metallographic polishing procedures and were etched by aqua-regia. The microstructures of the prepared samples were observed by optical microscope (OM) (Leica DM200, Wetzlar, Germany) and scanning electron microscopy (FEI Quanta 200, SEM, Eindhoven, The Netherlands) with back scattered electron (BSE) and energy dispersive spectroscopy (EDS) detector. To identify/assess the various phases present in the samples, X-Ray diffractometer (XRD, Panalytical, Almelo, The Netherlands) employing Cu-Kα radiation was used. Transmission electron microscope (TEM, Philips CM12, Eindhoven, The Netherlands) characterization was employed to study microstructure evolution during FS and to identify the second phase particles in the coated samples. For TEM studies, thin samples of 300 µm were sectioned transversely through EDM cutting machine. These thin samples were mechanically grounded to 100 µm and punched in to 3 mm disc. Then, these samples were twin-jet electro polished.

### 2.3. Mechanical Testing

The micro Vickers hardness was measured across the coating/substrate interface from the top surface of the Stellite 6 coating to the LCS base metal. A (Matsuzawa MMT-7, Akita-shi, Japan) digital microhardness tester was employed to conduct the experiments. The microhardness measurements were conducted at 500 g load applied for 15 s.

### 2.4. Wear Testing

**Figure 2:**
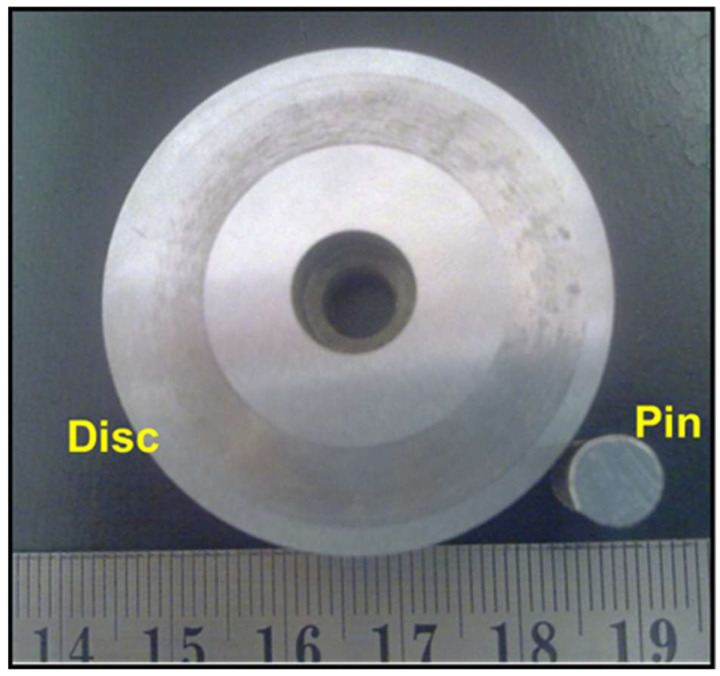
Pin-on-disc wear test set-up.

Dry sliding wear tests were carried out at room temperature using pin-on-disk equipment. Pin of 15 mm length and 6 mm diameter was prepared for all the above-mentioned material process conditions. A 56 mm disk of hardened alloy steel with a hardness of 64 HRC was used as the rotating disk for the machine. The disk was rotated at 650 rpm and load applied was 30 N. Each sample was characterized for sliding distance of 1000 m. These parameters were selected based on commonly used conditions in wear studies for hard-facing coatings and align with the ASTM G99 standard for pin-on-disk wear testing. Further, these parameters were chosen to simulate moderate contact stresses and sliding velocities encountered in real-world tribomechanical applications. Before the wear test, each sample was mechanically ground in SiC paper to an average surface roughness of about 0.8 µm and cleaned by using acetone in an ultrasonic bath for 15 min. The pin and disc after the wear test are shown in Figure 2.

## 3. Results and Discussion

### 3.1. Microstructures

#### 3.1.1. Substrate and Consumable Rod

**Figure 3:**
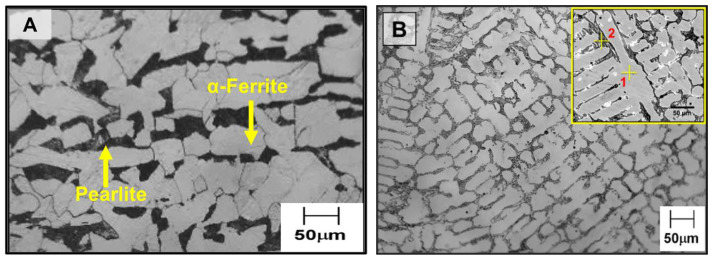
Optical micrographs of (**A**) hot rolled low carbon steel substrate and (**B**) cast Stellite 6 consumable rod. The inset in (**B**) shows the high magnification image with the EDS spot analysis location (1 and 2) indicated.

Figure 3A shows the optical micrograph of the LCS substrate comprising bands of α-ferrite and pearlite phases. Figure 3B indicates the microstructure of the cast Stellite 6 consumable rod, showing a coarse dendritic structure with γ-dendrites and an inter-dendritic carbide network. The results of the EDS spot analysis taken from the dendrite core (spot 1 in Figure 3B) and inter-dendritic regions (spot 2 in Figure 3B) of cast Stellite 6 are given in Table 4. The dendritic regions showed higher Co concentration than the inter-dendritic regions, and the inter-dendritic regions showed higher Cr than the dendrite core.

#### 3.1.2. Plasma Transferred Arc Coating

**Figure 4:**
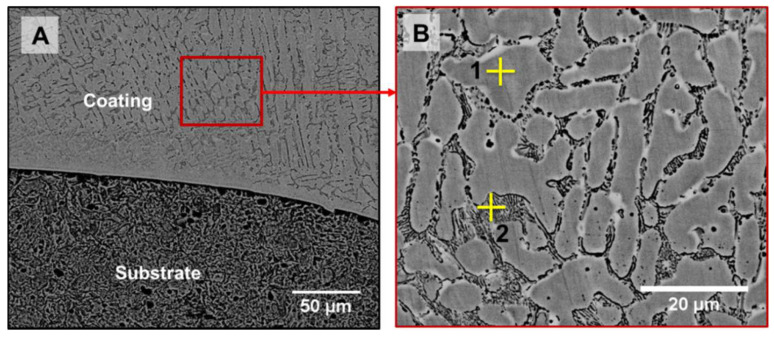
Microstructures of the PTA coating (**A**) at low magnification and (**B**) at higher magnification.

The microstructures of the PTA-deposited Stellite 6 coating are shown in Figure 4. PTA provides a solidified dendritic structure analogous to cast Stellite 6; however, the microstructures are much finer in the former compared to the latter. The average grain size in the HAZ of the PTA-coated sample is found to be 6 ± 1 µm. The EDS spot analysis on the dendrite core and inter-dendritic regions exhibits distinct Co and Cr concentrations; however, the difference is notably less than the cast Stellite 6. The EDS spot analysis results are listed in Table 5. The rapid cooling condition during the PTA process compared to the casting would provide less time for the elements to segregate to the inter-dendritic regions, thereby reducing segregation effects. Nevertheless, segregation is still significant in the PTA coatings.

#### 3.1.3. Friction Surfacing

**Figure 5:**
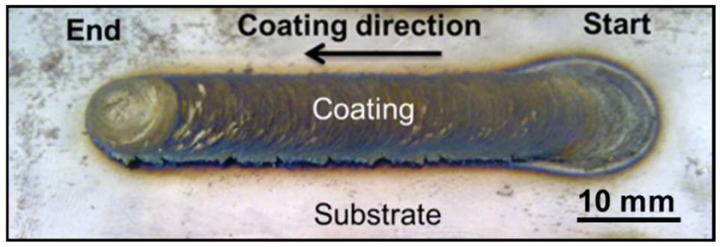
Photograph of friction surfaced Stellite 6 coating.

**Figure 6:**
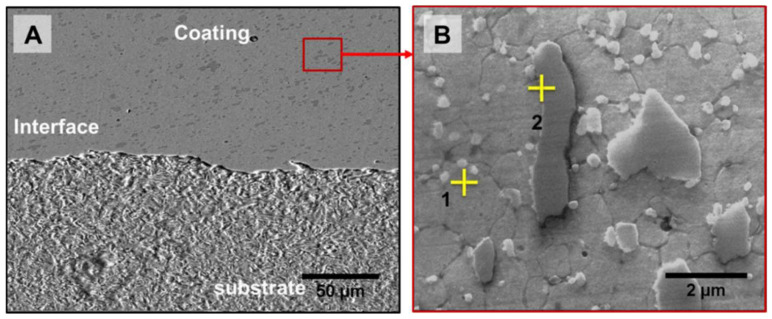
(**A**) SEM microstructures of friction surface-coated samples and (**B**) magnified view of the friction surface coating.

The photograph of the Stellite 6 coated sample prepared through FS is shown in Figure 5. Indeed, the Stellite 6 FS Coating is completely metallurgically bonded with the substrate LCS. The microstructures of the FS-coated sample and its interface are shown in Figure 6. The microstructure of the FS-coated sample does not exhibit any solidified structure, unlike the Stellite 6 consumable rod and PTA coating (Figure 3B and Figure 4B). Further, the interconnected carbide network at the inter-dendritic regions of the consumable rod is fragmented into extremely fine discrete particles, because of the severe plastic deformation of the consumable rod during FS. The average grain size of the FS-coated sample in the HAZ is 1 ± 0.5 µm. The frictional heating and the blending activity prompted a stream of the material in the whole surface of the bar, prompting the fracture of hard second stage/phase particles. The chemical composition of Co and Cr in the FS coating is mentioned in Table 6, which provides the evidence that composition homogeneity was achieved and different locations of Co or Cr were discovered to be homogeneous, demonstrating that FS produces compositional homogenization throughout the coated substrate [20].

### 3.2. Dilution Studies

**Figure 7:**
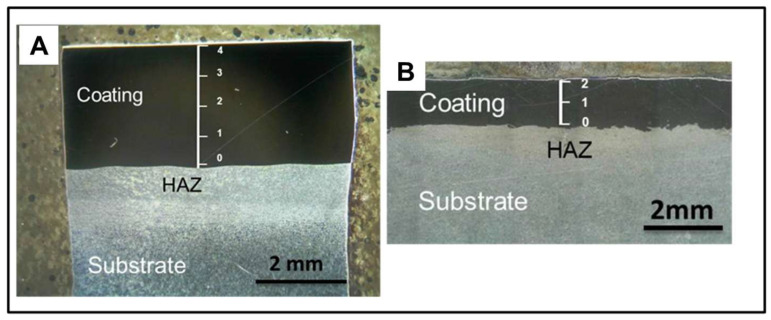
Macrograph images showing location of EDS spot analysis on PTA coatings (**A**) and FS coatings (**B**).

To determine the extent of dilution, the samples coated with Stellite 6 employing FS and PTA were subjected to the EDS examination as shown in Figure 7A and Figure 7B respectively. The average coating thicknesses of the FS- and PTA-coated samples are 1 ± 0.5 mm and 3 ± 0.5 mm, respectively, as shown in Figure 7A and Figure 7B. According to the EDS investigation, the amount of Co and Cr decreases as we approach the interface from the top surface of the coating, with a dilution of 15% for Co in PTA and just 1.6% in FS, and a dilution of 28.5% for Cr in PTA coated sample, and a negligible dilution for Cr in FS. Further, there is significant dilution of Fe from the base material. When compared from the extreme end of the coating to the base metals surface (which is closest to the HAZ), there is a case of enrichment of Fe in PTA. The Fe, which was originally 3 (Wt. %) at the extreme end of the coating, increases to 18 (Wt. %) at the base metals surface, which is an increase of six times. As we approach the HAZ, the Fe in the case of FS is diluted by 7.4% from the surface of the coating to the surface of the base metal.

### 3.3. Phase Analysis

**Figure 8:**
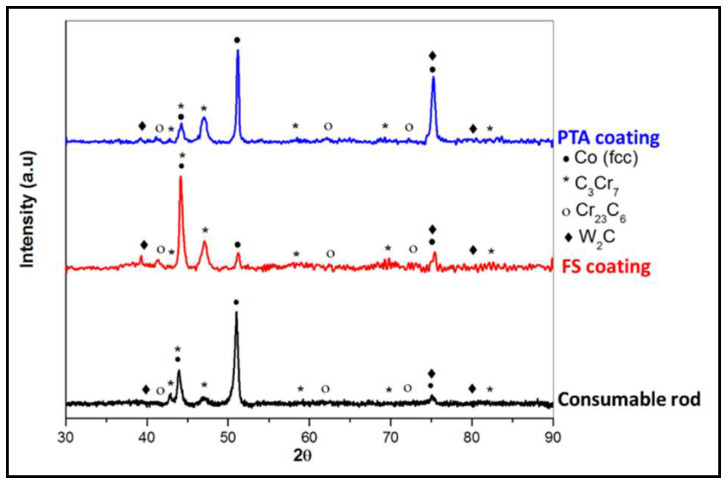
X-Ray diffraction patterns of the consumable rod, friction surfacing, and plasma transfer arc coatings.

**Figure 9:**
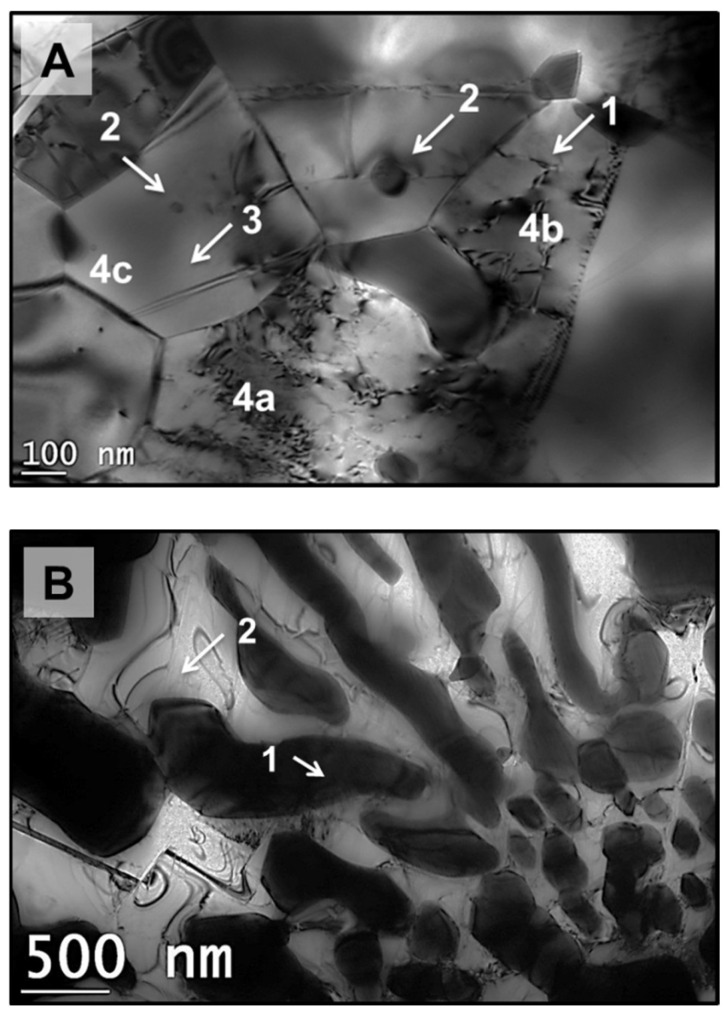
TEM microstructures of coated samples: (**A**) Friction surfacing, (**B**) Plasma Transfer Arc Coating. The annotations in (**A**): 1—dislocation sub-cell formation; 2—Fine carbide particles; 3—fine grain microstructure; and 4a, 4b, 4c—grains with low, medium, and high dislocation densities, respectively. The annotations in (**B**): 1—interconnected carbide network and 2—coarse dendritic structure.

The results of X-ray diffraction patterns obtained from FS, PTA, and the Stellite 6 consumable rod are shown in Figure 8. The phases found in the deposited coating are γ-Co (fcc) and carbides C_3_Cr_7_, Cr_23_C_6_, and W_2_C. The highest peak intensity is recorded for the γ-Co (fcc) and there is no Co-HCP phase identified in the FS and PTA coatings. Further, while the PTA and the Stellite 6 consumable rod showed a solidification texture (with highest intensity peak at <200> plane, <100> is the easy growth direction for cubic materials), the FS coatings showed no texture effects because of the severe plastic deformation leading to dynamic recrystallization. The occurrence of dynamic recrystallization in the FS coatings can be inferred from the TEM micrographs (Figure 9A). Equiaxed grains have varying low, medium, and high dislocation densities (indicated with 4a, 4b, and 4c, respectively, in Figure 9A) and dislocation sub-cell boundaries (indicated as 1 in Figure 9A) are present. Further, fine discrete carbide particles are seen in FS coatings, whereas a lamellar, interconnected carbide network is observed in the PTA coatings (Figure 9B).

### 3.4. Mechanical Properties

#### 3.4.1. Microhardness

**Figure 10:**
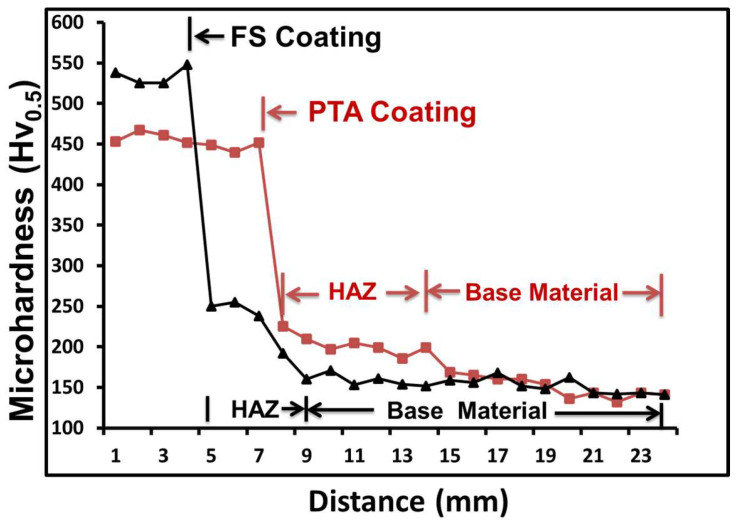
Micro Vickers hardness plots across the coating/substrate interface of the FS- and PTA-coated samples.

**Figure 11:**
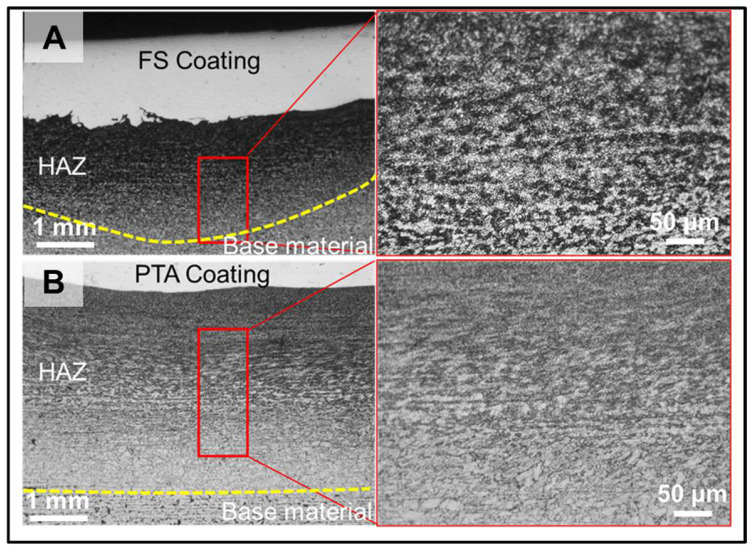
Optical microscope microstructures of the HAZ; (**A**) Friction surfacing and (**B**) PTA coatings.

Figure 10 depicts the hardness profile obtained from the vertical cross-section of the FS and PTA samples. As illustrated, the PTA coatings have a lower hardness of 450 HV compared to the 550 HV of the FS coating, demonstrating that the FS coatings perform better in terms of hardness than the PTA. The increased hardness in the FS coatings can primarily be attributed to the fine grain structure with uniform distribution of fine carbide particles in FS. This can be noted clearly from Figure 11A. Importantly, the hardness increases in the HAZ to a larger width in the PTA-coated samples, ~6 mm, compared to the FS-coated samples, ~1 mm (Figure 11). This indicates that the width of the HAZ during FS will be significantly less compared to the PTA coatings. The difference in heat input between the two processes has mostly contributed to the HAZ being substantially thicker in the case of PTA compared with FS. It is highly desirable to minimize the HAZ as much as possible because it has adverse effects on the base metal strength and toughness [31]. It is worth noting that although with no preheating in FS, the HAZ hardness in the FS is similar to PTA samples. During FS, the peak temperature was reported to be ~0.8 T_m_, and the cooling rates were rapid (with no preheating) [28]. These conditions resulted in lower HAZ width in the FS samples compared to PTA coatings (which experienced peak temperatures greater than the melting point of the alloy with high preheating temperatures of about ~300 °C).

#### 3.4.2. Wear Characterization

**Figure 12:**
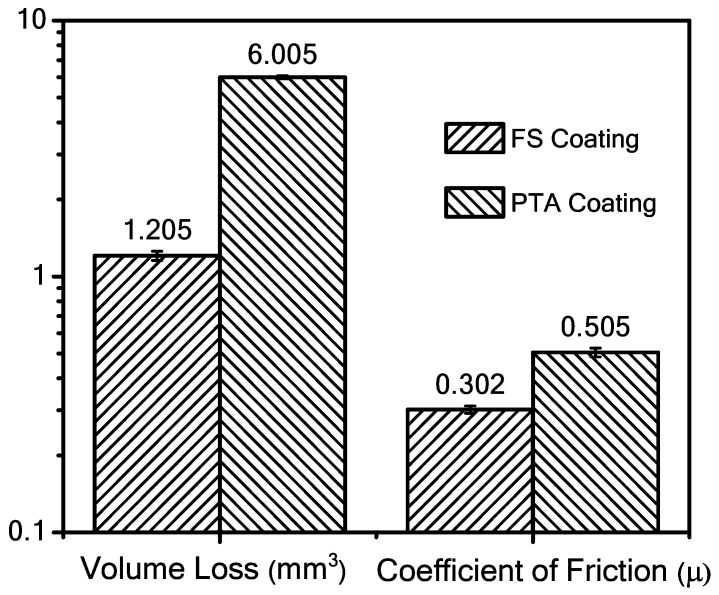
Results of pin-on-disc wear test at room temperature for the FS and PTA coatings.

The pin-on-disc wear test results of FS and PTA Stellite 6 coatings are presented in Figure 12. Here, two parameters, volume loss (mm^3^) and coefficient of friction (µ), are compared. With the FS coating, the volume loss is 1.205 mm^3^, and it is 6.005 mm^3^ with PTA. It is evident that FS has significantly less volume loss than PTA, primarily because the FS sample possesses higher hardness comparatively. Additionally, the coefficient of friction is 0.505 and 0.302 for PTA and FS samples, respectively. In other words, the higher the coefficient of friction, the more quickly that metal will wear [6,7,9,11,21,22,23,32,33]. The FS approach is superior to PTA when comparing the two aforementioned characteristics. As mentioned in [34], the resistance to abrasive and adhesive wear is seen in both FS and PTA coatings due to the presence of hard carbide particles. Additionally, FS coating outperforms PTA since it has more hardness, meaning no further work hardening is possible, which is mainly attributed to the grain refinement from fine grains. It was observed that the wear resistance of FS coating increases as the material resistance to plastic deformation increases. However, more plastic deformation is seen in the case of PTA.

**Figure 13:**
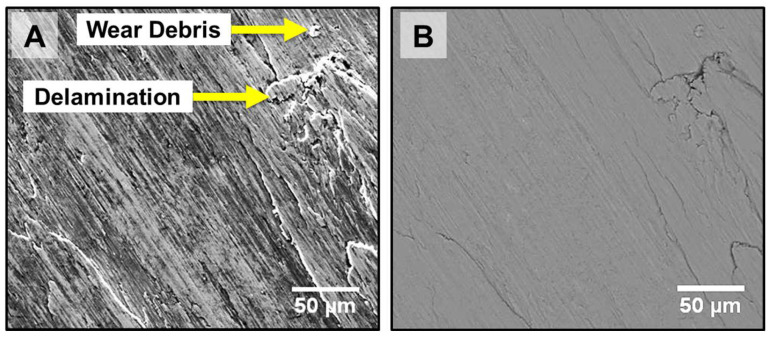
Surface morphology of PTA-coated sample after wear test: (**A**) SEM-SE Image, (**B**) SEM-BSE Image.

Figure 13 represents the surface morphology of the PTA sample after the wear test. There are ledge areas and delamination formed as shown in Figure 13A, which are indications of plastic deformation. It should be emphasized that the accumulation of dislocations beneath the wear surface is the primary cause of the wear lamellas. Dislocations begin to coalesce close to the surface during sliding contact as seen in Figure 13B. Small cavities will occur in such regions because the production of voids and cavities in regions with a high density of dislocations reduces the energy [35]. As voids continue to expand as a result of applied stresses, they eventually reach a critical length where they can start to crack. The wear particles separate off the surface in the form of a thin film due to the convergence of these cracks.

**Figure 14:**
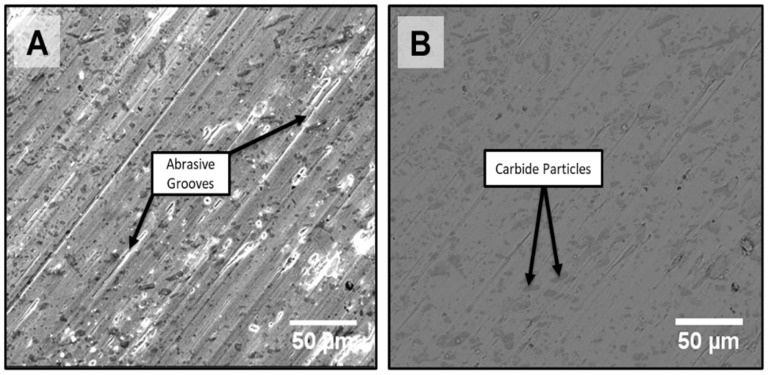
Surface morphology of FS-coated sample after wear test; (**A**) SEM-SE Image, (**B**) SEM-BSE Image.

Figure 14 shows the surface morphology of the FS sample after the wear test. It can be observed that there is fine distribution of carbide particles throughout the matrix as shown in Figure 14B with abrasive grooves seen in Figure 14A; this formation of high-density coatings led to a drastic increase in obtained hardness values with a reduction in the coefficient of friction value; this is a significant factor that shows that FS has greater wear resistance than PTA. The relationship between the change in wear rate and the change in coating hardness holds true for all coatings. Consequently, increasing the coating hardness is necessary to reduce the wear rate. According to [36], the hardness and wear rate are inversely related. Due to the presence of fine dispersion of carbide particles, equiaxed grains exhibit improved wear properties. Further, an adhesive wear mechanism is observed in the worn surface of the FS-coated samples. It can be seen that, as the hardness of the material decreases, the depth and width of the grooves increases, as does the amount of the abrasive mechanism.

The FS Stellite 6 coatings in the current study exhibit superior wear resistance compared to PTA coatings and previously reported results. The volume loss of FS coatings (1.205 mm^3^) is significantly lower than PTA (6.005 mm^3^) and those reported in [13] at a much lesser load (15 N), highlighting the enhanced performance at higher loads. Similarly, the coefficient of friction for FS (0.302) is lower than PTA (0.505) and the values reported by Magarò et al. [37] (0.4–0.65 at low speeds), suggesting better tribological properties. Yoon et al. [38] found that dilution negatively affects Stellite 6 wear resistance, while our FS coatings, free from dilution effects, demonstrate improved hardness and resistance to plastic deformation. These findings indicate that FS coatings offer a more effective wear-resistant solution compared to conventional PTA methods. The worn surface morphologies in the current study indicate abrasive and adhesive wear mechanisms in both FS and PTA coatings. Similar findings were reported by Magaro et al. [38], who observed subsurface cracking and plastic deformation in Stellite 6 coatings under reciprocating dry sliding conditions [38]. However, in the present study, FS coatings exhibited finer carbide dispersion and reduced plastic deformation, leading to improved wear performance.

## 4. Conclusions

- The FS and PTA coatings exhibited excellent bonding at the substrate/coating interface. The FS coatings developed a wrought microstructure with dynamically recrystallized grains containing fine, uniformly distributed carbides, while the PTA coatings showed a cast microstructure with coarse γ-dendrites and an inter-dendritic carbide network.
- Dilution and chemical uniformity varied between the two processes. PTA coatings exhibited significant dilution and localized compositional variations, whereas FS coatings achieved a more uniform composition across the thickness with minimal dilution.
- The HAZ in FS coatings (~1 mm) was significantly narrower than in PTA coatings (~6 mm), indicating that the solid-state nature of FS processing leads to reduced heat input and more controlled thermal cycles.
- The hardness of FS coatings was superior to that of PTA coatings, which is attributed to the formation of dynamically recrystallized grains with finely dispersed carbide particles.
- Wear resistance testing demonstrated that FS coatings (1.205 mm^3^ wear loss) exhibited significantly better wear resistance compared to PTA coatings (6.005 mm^3^ wear loss). The improved performance is primarily due to the refined microstructure and higher hardness in FS coatings.

In summary, this study demonstrates that FS is a viable and effective alternative to PTA deposition for Stellite 6 coatings on LCS substrates. The superior microstructural refinement, reduced dilution, and enhanced hardness of FS coatings result in significantly improved wear resistance, making FS a promising process for applications requiring wear-resistant coatings. These findings highlight the potential of FS for industrial applications such as aerospace, automotive, and tooling industries, where high-performance wear coatings are critical. Future studies can explore the optimization of FS process parameters and FS’s applicability to other coating materials for broader engineering applications.

## Figures and Tables

**Table 1:** Chemical composition of the Stellite 6 consumable rod and LCS substrate.

Materials	Composition (Wt%)								
	Cr	W	C	Fe	Co	Si	Mn	P	S
Stellite 6 consumable rod	27.50	5.00	3.30	2.90	bal.	-	-	-	-
LCS substrate	-	-	0.12	bal.	-	0.36	0.42	0.02	0.01

**Table 2:** Process parameters used for the PTA process.

Current (A)	100
Weld Speed (mm/s)	1.20
Polarity	DCEN
Oscillation width (mm)	10
Preheating (°C)	300
No. of layers	2
Powder feed rate (g/min)	30
Shielding gas(Ar) flow rate (L/min)	12
Powder feed gas flow rate (L/min)	3.5
Torch work distance (mm)	15
Plasma gas(Ar) flow rate (L/min)	4

**Table 3:** Process parameters used for the FS process.

Spindle speed (rpm)	800
Axial/Vertical load (kN)	0.9
Substrate transverse feed (mm/s)	1.6
Dwell time (s)	30

**Table 4:** EDS spot analysis results of the Stellite 6 consumable rod. The EDS spot analysis locations (1 and 2) are indicated in the inset of Figure 3B.

Location	Composition (Wt.%)			
	Co	Cr	W	Fe
Location—1 (dendrite Core)	63	24	7	5
Location—2 (inter-dendritic region)	36	50	9	4

**Table 5:** EDS spot analysis on the PTA coating. The EDS spot analysis locations (1 and 2) are indicated in Figure 4B.

Location	Co	Cr
Location 1 (dendrite core)	56	22
Location 2 (inter-dendritic region)	45	37

**Table 6:** EDS spot analysis on PTA coatings and FS coatings at different locations.

Location	PTA Coating (Wt. %)			FS Coating (Wt. %)		
	Co	Cr	Fe	Co	Cr	Fe
4	60	28	3			
3	58	26	5			
2	55	24	12	61	27	2.7
1	54	22	15	61	28	2.6
0	51	20	18	60	27	2.5

## Data Availability

The original contributions presented in this study are included in the article. Further inquiries can be directed to the corresponding authors.

## Abbreviations

The following abbreviations are used in this manuscript:

FS	Friction Surfacing
PTA	Plasma Transferred Arc
LCS	Low Carbon Steel
GTAC	Gas Tungsten Arc Coating
HVOF	High Velocity Oxygen Fuel
SEM	Scanning Electron Microscopy
XRD	X-Ray Diffractometer
EDS	Energy Dispersive Spectroscopy
TEM	Transmission Electron Microscope
EDM	Electrical Discharge Machining
HAZ	Heat Affected Zone

## References

[1] Singh R. (2016). Applied Welding Engineering: Processes, Codes, and Standards.

[2] Rao K.P., Damodaram R., Rafi H.K., Ram G.J., Reddy G.M., Nagalakshmi R. (2012). Friction surfaced Stellite6 coatings. Mater. Charact..

[3] Corchia M., Delogu P., Nenci F., Belmondo A., Corcoruto S., Stabielli W. (1987). Microstructural aspects of wear-resistant stellite and colmonoy coatings by laser processing. Wear.

[4] D’Oliveira A.S.C.M., Da Silva P.S.C.P., Vilar R.M.C. (2002). Microstructural features of consecutive layers of Stellite 6 deposited by laser cladding. Surf. Coat. Technol..

[5] Sidhu B.S., Prakash S. (2006). Erosion-corrosion of plasma as sprayed and laser remelted Stellite-6 coatings in a coal fired boiler. Wear.

[6] Vite M., Castillo M., Hernández L.H., Villa G., Cruz I.H., Stéphane D. (2005). Dry and wet abrasive resistance of Inconel 600 and stellite. Wear.

[7] Shin J.C., Doh J.M., Yoon J.K., Lee D.Y., Kim J.S. (2003). Effect of molybdenum on the microstructure and wear resistance of cobalt-base Stellite hardfacing alloys. Surf. Coat. Technol..

[8] Malayoglu U., Neville A., Lovelock H. (2005). Assessing the kinetics and mechanisms of corrosion of cast and HIPed Stellite 6 in aqueous saline environments. Corros. Sci..

[9] Lima C.R.C., Belém M.J.X., Fals H.D.C., Rovere C.A.D. (2020). Wear and corrosion performance of Stellite 6^®^ coatings applied by HVOF spraying and GTAW hotwire cladding. J. Mater. Process. Technol..

[10] Sidhu T.S., Prakash S., Agrawal R.D. (2006). Hot corrosion studies of HVOF NiCrBSi and Stellite-6 coatings on a Ni-based superalloy in an actual industrial environment of a coal fired boiler. Surf. Coat. Technol..

[11] Madadi F., Shamanian M., Ashrafizadeh F. (2011). Effect of pulse current on microstructure and wear resistance of Stellite6/tungsten carbide claddings produced by tungsten inert gas process. Surf. Coat. Technol..

[12] Singh P.K., Mishra S.B. (2020). Studies on solid particle erosion behaviour of D-Gun sprayed WC-Co, Stellite 6 and Stellite 21 coatings on SAE213-T12 boiler steel at 400 °C temperature. Surf. Coat. Technol..

[13] Zhu Z., Ouyang C., Qiao Y., Zhou X. (2017). Wear Characteristic of Stellite 6 Alloy Hardfacing Layer by Plasma Arc Surfacing Processes. Scanning.

[14] Cinca N., Guilemany J.M. (2013). Cold Gas Sprayed Stellite-6 Coatings and their Wear Resistance. J. Mater. Sci. Eng..

[15] Wei L., Dong Y., Zhao Y., Xu Z., Li H., Wu Y. (2024). The Influence of Ti and Ni on the Microstructure, Wear Resistance, and Thermal Fatigue Performance of Laser Cladded Stellite 6 Coatings. J. Mater. Eng. Perform..

[16] Félix-Martínez C., Salgado-López J.M., López-Martínez A., García-Salas L.D., González-Carmona J., Cruz-González C.E. (2024). Microstructure; hardness, and wear resistance at room and high temperature of Stellite-6/WC-6Co coatings deposited by laser cladding process. Int. J. Adv. Manuf. Technol..

[17] Manukonda S., Bijjam R.R. (2023). Wear Resistance of Stellite-6/TiC Coating on Stainless Steel 316L Produced by Laser Cladding Process. Ann. Chim. Sci. Mater..

[18] Moradi M., Ashoori A., Hasani A. (2020). Additive manufacturing of stellite 6 superalloy by direct laser metal deposition—Part 1: Effects of laser power and focal plane position. Opt. Laser Technol..

[19] Nair A., Ramji V., Raj R.D., Veeramani R. (2020). Laser cladding of Stellite 6 on EN8 steel—A fuzzy modelling approach. Mater. Today Proc..

[20] Nair A., Khan A. (2020). Studies on effect of laser processed stellite 6 material and its electrochemical behavior. Optik.

[21] Rajeev G.P., Rahul M.R., Kamaraj M., Bakshi S.R. (2020). Microstructure and high temperature mechanical properties of wire arc additively deposited Stellite 6 alloy. Materialia.

[22] Singh J., Kumar S., Mohapatra S.K. (2020). Erosion tribo-performance of HVOF deposited Stellite-6 and Colmonoy-88 micron layers on SS-316L. Tribol. Int..

[23] Thawari N., Gullipalli C., Katiyar J.K., Gupta T.V.K. (2021). Influence of buffer layer on surface and tribomechanical properties of laser cladded Stellite 6. Mater. Sci. Eng. B Solid State Mater. Adv. Technol..

[24] Wang G., Zhang J., Shu R., Yang S. (2019). High temperature wear resistance and thermal fatigue behavior of Stellite-6/WC coatings produced by laser cladding with Co-coated WC powder. Int. J. Refract. Met. Hard Mater..

[25] Moradi M., Hasani A., Beiranvand Z.M., Ashoori A. (2020). Additive manufacturing of stellite 6 superalloy by direct laser metal deposition—Part 2: Effects of scanning pattern and laser power reduction in differrent layers. Opt. Laser Technol..

[26] Sidhu H.S., Sidhu B.S., Prakash S. (2007). Solid particle erosion of HVOF sprayed NiCr and Stellite-6 coatings. Surf. Coat. Technol..

[27] Sidhu B.S., Puri D., Prakash S. (2005). Mechanical and metallurgical properties of plasma sprayed and laser remelted Ni-20Cr and Stellite-6 coatings. J. Mater. Process. Technol..

[28] Rajoria V., Nain G., Vijayan S., Prasad C.H., Damodaram R., Karthik G.M., Khan F. (2022). Materials Today: Proceedings Development of SS 304L composite coatings on mild steel substrate using friction surfacing and wear characterization. Mater. Today Proc..

[29] Khan M.D.F., Rokkala U. (2023). Development of high strength and corrosion resistance Mg-Zn-Dy/HA-Ag composite for temporary implant applications. Mater. Lett..

[30] Sadhu A., Karmakar D.P., Mypati O., Muvvala G., Pal S.K., Nath A.K. (2020). Performance of additive manufactured Stellite 6 tools in friction stir processing of CuCrZr sheet. Opt. Laser Technol..

[31] Silwal B., Li L., Deceuster A., Griffiths B. (2013). Effect of Postweld Heat Treatment on the Toughness of Heat-Affected Zone for Grade 91 Steel. Weld. Res..

[32] Opris C.D., Liu R., Yao M.X., Wu X.J. (2007). Development of Stellite alloy composites with sintering/HIPing technique for wear-resistant applications. Mater. Des..

[33] Ramachandran C.S., Balasubramanian V., Varahamoorthy R. (2010). Comparative evaluation of dry sliding wear behaviour of plasma transferred arc hardfaced surfaces by the pin-on-roller method. Proc. Inst. Mech. Eng. Part J J. Eng. Tribol..

[34] Cao H.T., Dong X.P., Pan Z., Wu X.W., Huang Q.W., Pei Y.T. (2016). Surface alloying of high-vanadium high-speed steel on ductile iron using plasma transferred arc technique: Microstructure and wear properties. Mater. Des..

[35] Mousavi S.E., Naghshehkesh N., Amirnejad M., Shammakhi H., Sonboli A. (2021). Wear and Corrosion Properties of Stellite-6 Coating Fabricated by HVOF on Nickel–Aluminium Bronze Substrate. Met. Mater. Int..

[36] Rahmati Z., Aval H.J., Nourouzi S., Jamaati R. (2022). Effect of copper reinforcement on the microstructure, macrotexture, and wear properties of a friction-surfaced Al-Cu-Mg coating. Surf. Coat. Technol..

[37] Magarò P., Furgiuele F., Maletta C., Tului M., Wood R.J.K. (2023). Wear Mechanisms of Cold-Sprayed Stellite-6 During Reciprocated Dry Sliding Under Different Sliding Speeds. J. Therm. Spray Technol..

[38] Yoon B.H., Lee C.H., Kim H.J. (2017). Effect of Dilution on wear performance of plasma transferred Arc deposited layers. ISIJ Int..

